# “What do you think about nephrology?” A national survey of internal medicine residents

**DOI:** 10.1186/s12882-021-02397-9

**Published:** 2021-05-21

**Authors:** Georges N. Nakhoul, Ali Mehdi, Jonathan J. Taliercio, Susana Arrigain, Jesse D. Schold, Abby Spencer, Jessica Greenfield, Amit Diwakar, Grace Snyder, John O’Toole, Joseph V. Nally, John R. Sedor, Patricia F. Kao, S. Beth Bierer

**Affiliations:** 1 Department of Hypertension and Nephrology, Glickman Kidney Urological Institute, Cleveland Clinic Foundation, Cleveland, OH USA; 2 Cleveland Clinic Lerner College of Medicine of Case Western Reserve University, Cleveland, OH USA; 3 Department of Quantitative Health Sciences, Cleveland Clinic Foundation, Cleveland, OH USA; 4 Department of Internal Medicine, Medicine Institute, Cleveland Clinic Foundation, Cleveland, OH USA; 5 Education institute, Cleveland Clinic Foundation, Cleveland, OH USA; 6 Department of Internal Medicine, Cleveland Clinic Akron General Hospital, Akron, OH USA; 7 Department of Internal Medicine, Texas Institute for Graduate Medical Education and Research Hospital, Laredo, OH USA; 8 Department of Nephrology, Washington University in Saint Louis, Saint Louis, MO USA

**Keywords:** Nephrology fellowship, Specialty training, Education

## Abstract

**Background:**

Interest in nephrology has been declining among internal medicine residents but the reasons behind this observation are not well characterized. Our objective was to evaluate factors influencing residents’ choice of subspecialty.

**Methods:**

This is a mixed-method QUAL-QUAN design study that used the results of our previously published qualitative analysis on residents’ perception of nephrology to create and pilot a questionnaire of 60 questions. The final questionnaire was distributed to 26 programs across the United States and a total of 1992 residents. We calculated response rates and tabulated participant characteristics and percentage of participant responses. We categorized choice of fellowship into 2 medical categories (Highly Sought After vs. Less Sought After) and fitted a logistic regression model of choosing a highly vs. less sought after fellowship.

**Results:**

Four hundred fifteen out of 1992 (21%) US residents responded to the survey. Of the 268 residents planning to pursue fellowship training, 67 (25%) selected a less sought after fellowship. Female sex was associated with significantly higher odds of selecting a less sought after fellowship (OR = 2.64, 95% CI: 1.47, 4.74). Major factors deterring residents from pursuing nephrology were perception of inadequate financial compensation, broad scope of clinical practice and complexity of patient population. We observed a decline in exposure to nephrology during the clinical years of medical school with only 35.4% of respondents rotating in nephrology versus 76.8% in residency. The quality of nephrology education was rated less positively during clinical medical school years (median of 50 on a 0–100 point scale) compared to the pre-clinical years (median 60) and residency (median 75).

**Conclusion:**

Our study attempts to explain the declining interest in nephrology. Results suggest potential targets for improvement: diversified trainee exposure, sub-specialization of nephrology, and increased involvement of nephrologists in the education of trainees.

**Supplementary Information:**

The online version contains supplementary material available at 10.1186/s12882-021-02397-9.

## Introduction

Residents’ interest in a nephrology career has been steadily declining over the last decade as indicated by the decreasing number of applicants to the specialty. Whereas 578 applicants applied to 367 positions in the United States in 2009, only 330 applied in 2019 to 469 positions. As a result, 58.6% of nephrology programs and 38% of nephrology positions remained unfilled in 2019 [1]. This decrease in interest appears even more pronounced among United States Medical Graduates (USMGs), with only 27% of nephrology fellows coming from US allopathic medical school, and 19% from osteopathic medical schools [2]. This waning popularity in nephrology as a subspecialty is highly concerning given the concomitant increasing importance of the subspecialty in the healthcare of the American population. Indeed, the rising prevalence of Chronic Kidney Disease (CKD) and End-Stage Kidney Disease (ESKD) in the United States [3] (US) is being met with a shrinking nephrology workforce, and this has generated widespread concern about the future of the profession [4].

In response to this crisis, the American Society of Nephrology (ASN) created a task force charged with increasing interest in nephrology careers. This endeavor has generated several initiatives that provided analyses of job markets and of factors influencing interest in the specialty. In particular, the findings of the task force provided vital data on specialty perceptions by nephrology fellows, practicing physicians, and large dialysis organizations [5]. However, the task force did not study US internal medicine residents, whose attitudes and opinions are key as they constitute the nephrology’s major pipeline. Additionally, among the rare studies looking at nephrology perceptions among medicine residents, the vast majority obtained data via questionnaires that were designed by the investigators, without input from the residents [6–8]. Questions posed to study participants in these studies are therefore subject to the personal biases and values of the investigators. Only one study used focus groups to inform the survey questions [9]; none explained the theoretical framework in which investigations were grounded.

In order to address this gap, we built upon our published qualitative study that detailed factors influencing residents’ perceptions of nephrology [10] and used our findings to design a survey of 60 questions. We recruited the participation of 26 programs across the United States. Our aims were to understand factors influencing internal medicine residents’ choice of the 8 major medicine subspecialties (cardiology, endocrinology, infectious disease, gastroenterology, nephrology, hematology-oncology, pulmonary-critical care and rheumatology), and in particular, to gain insight on their perceptions of nephrology.

## Material and methods

### Research design

In our prior study, we conducted a qualitative assessment that highlighted the factors influencing internal medicine residents’ decisions to pursue specialty training along with their perceptions of nephrology [10]. The study consisted of semi-structured interview questions that were conducted using the Professional Identity Formation (PIF) framework [11], while the data analysis was guided by the content analysis approach [12]. Using our results, we then designed a questionnaire draft that was based on the themes reported by the residents. The questionnaire was pilot-tested by a multi-institutional group consisting of five nephrology faculty, five nephrology and post-doctoral fellows and four internal medicine chief residents. Each individual was invited to take the survey and provide their feedback separately. The survey was revised according to the collective suggestions and then sent again to the same individuals for a second round of piloting. After a second round of revisions, the survey was piloted a third time in a focus group that included faculty members and finalized based on their consensus. The final version consisted of 60 multiple-choice, yes/no, open-response questions and question rating on a 0 to 100 scale, that were divided into the following five categories: demographics, current training, career plans, exposure to nephrology, and nephrology perceptions. The survey allowed the respondents to skip the questions they preferred not to answer. It was anonymous and was delivered using the secure web application “REDCap”. The study was reviewed by the Cleveland Clinic Internal Review Board (IRB) committee and was deemed exempt. All methods were performed in accordance with the relevant and recognized guidelines and regulations.

### Study participants

The survey was conducted between January and June of 2020. Using the Accreditation Council for Graduate Medical Education (ACGME) public website, we identified and invited all accredited internal medicine programs (*n* = 569) by contacting either the program director or the program coordinator (when the program director email was not listed). If the program agreed to participate in the study, the survey link was sent to the point of contact within the program. The point of contact then forwarded the survey link to the residents in their program. We requested that three additional reminders be issued on a weekly basis. In order to encourage participation, we included the possibility to win $50 (2–6 winners per program based on the size of the program). In total, 26 residency programs across 16 different states agreed to participate in the study. The survey was distributed to 1992 internal medicine residents. A list of programs is provided in Table 1.

**Table 1:** Programs participating in the survey

*Residency Program*	*State*	*N response*	*N in program*	*Percent Response*
University of Arizona, Tucson	Arizona	18	85	21.2
University of Colorado, Denver	Colorado	5	150	3.3
University of North Dakota	North Dakota	1	24	4.2
Cleveland Clinic Florida	Florida	9	32	28.1
Mayo Clinic, Jacksonville	Florida	5	52	9.6
WellStar Atlanta Medical Center	Georgia	2	30	6.7
Michigan State University / Sparrow Hospital	Michigan	19	45	42.2
Saint Louis University	Missouri	22	75	29.3
Washington University of St. Louis	Missouri	51	128	39.8
University of North Carolina Chapel Hill	North Carolina	22	95	23.2
SUNY Upstate Medical University	New York	27	131	20.6
Cleveland Clinic main campus	Ohio	61	165	37.0
Cleveland Clinic Akron	Ohio	15	36	41.7
Cleveland Clinic Fairview	Ohio	3	38	7.9
Metrohealth systems	Ohio	12	66	18.2
University of Toledo	Ohio	3	60	5.0
University Hospitals Cleveland Medical Center	Ohio	49	140	35.0
Wright State University	Ohio	12	75	16.0
University of Tennessee	Tennessee	10	90	11.1
Vanderbilt University Medical Center	Tennessee	26	139	18.7
TIGMER	Texas	4	17	23.5
Texas Tech University (Permian Basin)	Texas	2	43	4.7
University of Virginia Medical Center	Virginia	18	101	17.8
Madigan Army Medical Center	Washington	3	35	8.6
Gundersen Lutheran Medical Center	Wisconsin	2	24	8.3
West Virginia University	West Virginia	13	69	18.8

### Data analysis

We tabulated the characteristics of the programs participating in the survey including number of residents, program type (community vs. university based), and whether the program was a tertiary referral hospital.

We calculated the percent response among those offered the survey. Based on those that responded, we tabulated participant characteristics, and calculated the percentage of participants selecting each response to each survey question. We created boxplots of the participant-rated, quality of nephrology education at different stages of medical education: pre-clinical medical school years, clinical medical school years, and residency. We also created lollipop plots to show the percent of respondents selecting responses about their training and views on nephrology and fellowship overall and by gender.

Among participants choosing the 8 medical fellowships evaluated in the survey (cardiology, gastroenterology, hematology-oncology, pulmonary-critical care, nephrology, rheumatology, infectious diseases, and endocrinology), we used Chi-square and Fisher’s exact tests to evaluate whether residents who had a particular specialty rotation during medical school had a higher proportion choosing that specialty compared to those who did not complete the rotation. Fisher’s Exact Test was used when expected cell counts of less than 5 comprised 25% or more of a table. In this subset of participants we also used generalized estimating equations with an independent correlation structure to evaluate the association between completing a nephrology rotation as a medical student vs. completing a rotation on any of the other specialties with choosing that specialty while including all rotations completed by each participant. We evaluated several correlation structures for the model and selected the one with the best fit according to the QIC (Quasi-likelihood under the independence model criterion).

To evaluate the association between completing a specialty rotation as a medical student and choosing that specialty for fellowship, we used generalized estimating equations with an independent correlation structure. We fitted this model with all study participants (including those that didn’t choose a fellowship) and all possible rotations of interest for each participant (cardiology, gastroenterology, hematology-oncology, pulmonary-critical care, nephrology, rheumatology, infectious diseases, and endocrinology).

We categorized choice of fellowship into 2 categories: Highly Sought After and Less Sought After. Our choice was based on examining the distribution of residents’ responses, whereas the four fellowships included in the highly sought after category (cardiology, gastroenterology, hematology-oncology and pulmonary-critical care) were sought by more than two thirds of the applicants. We fitted a logistic regression model of choosing a highly sought after vs. less sought after fellowship that evaluated the following variables: age < 30 vs. > 30, sex, white race vs. others, graduate of an US medical school vs. all others, and US citizenship vs. other.

## Results

Table 1 shows the response rate at each program participating in the survey. Appendix Table 1 shows the characteristics of the programs participating in the survey.

A total of 415 out of 1992 US residents responded to the survey, which corresponds to a response rate of 21%. Of these, 57% were male, 78% were US citizens and 72% graduated from US medical schools. The demographic characteristics of the responders are displayed in Table 2.

**Table 2:** Respondent characteristics

Factor	Total (***N*** = 415)
Age	
< 25	3 (0.73)
25–29	256 (62.1)
30–34	135 (32.8)
> 34	18 (4.4)
Gender	
Male	233 (56.6)
Female	178 (43.2)
Other	1 (0.24)
PGY year	
1	142 (34.5)
2	128 (31.1)
3	126 (30.6)
Other	16 (3.9)
Race/Ethnicity	
African American	21 (5.1)
Asian: East Asian	26 (6.3)
Asian: South East Asian	74 (18.0)
Caucasian	208 (50.7)
Hispanic/Latino	26 (6.3)
Other	55 (13.4)
School	
US Allopathic Medical School	251 (61.1)
US Osteopathic Medical School	45 (10.9)
International Medical School (Caribbean)	28 (6.8)
International Medical School (other than Caribbean)	87 (21.2)
Nationality	
US citizen	320 (77.9)
US permanent resident	11 (2.7)
H1/H2/H3 visa (temporary worker)	27 (6.6)
J1/J2 visa (exchange worker)	52 (12.7)
Other	1 (0.24)
Type of program	
University-based program	343 (82.7)
Community-based program	61 (14.7)
Unsure	11 (2.7)
Size of hospital	
< 200 beds	3 (0.73)
200–500 beds	93 (22.5)
> 500 beds	278 (67.3)
Unsure	39 (9.4)
Tertiary/Referral center	
Yes	365 (88.6)
No	20 (4.9)
Unsure	27 (6.6)

Statistics presented as N (column %)

### Highly sought after vs. less sought after fellowships

Two hundred ninety-five residents (71.6%) expressed interest in pursuing fellowship training. The most highly sought after fellowships were cardiology (22.4%) and gastroenterology (16.3%). Nephrology was among the less sought after fellowships, attracting only 6.4% of respondents (Table 3 and Table 4). Among 268 participants that selected one of the 8 fellowships of interest, 67 (25%) selected a less sought after fellowship. In the model including participant characteristics, female sex was associated with significantly higher odds of selecting a less sought after fellowship (OR = 2.64, 95% CI: 1.47, 4.74) (Table 5).

**Table 3:** Completing rotation during clinical years of medical school vs. choosing fellowship specialty

Rotations as a Medical Student	No (*N* = 202)	Cardiology (*N* = 66)	*p*-value
Cardiology			0.006^c^
No	84 (84.8)	15 (15.2)	
Yes	118 (69.8)	51 (30.2)	
	No (*N* = 220)	GI (*N* = 48)	*p-value*
Gastroenterology			*< 0.001*^*c*^
No	131 (91.6)	12 (8.4)	
Yes	89 (71.2)	36 (28.8)	
	No (*N* = 224)	Hemato/Oncology (*N* = 44)	*p-value*
Hemato/Oncology			*< 0.001*^*c*^
No	167 (92.8)	13 (7.2)	
Yes	57 (64.8)	31 (35.2)	
	No (*N* = 225)	Pulmonary/Critical (*N* = 43)	*p-value*
Pulmonary/Critical Care			*0.007*^*c*^
No	96 (91.4)	9 (8.6)	
Yes	129 (79.1)	34 (20.9)	
	No (*N* = 244)	Rheumatology (*N* = 24)	*p-value*
Rheumatology			*0.003*^*d*^
No	208 (93.7)	14 (6.3)	
Yes	36 (78.3)	10 (21.7)	
	No (*N* = 249)	Nephrology (*N* = 19)	*p-value*
Nephrology			*0.003*^*c*^
No	164 (96.5)	6 (3.5)	
Yes	85 (86.7)	13 (13.3)	
	No (*N* = 255)	ID (*N* = 13)	*p-value*
Infectious disease			*0.004*^*c*^
No	161 (98.2)	3 (1.8)	
Yes	94 (90.4)	10 (9.6)	
	No (*N* = 257)	Endocrinology (*N* = 11)	*p-value*
Endocrinology			*0.24*^*d*^
No	206 (96.7)	7 (3.3)	
Yes	51 (92.7)	4 (7.3)	

Statistics presented as N (row %)

*p*-values: ^c^Pearson’s chi-square test, ^d^Fisher’s exact test

Test comparing choosing Nephrology fellowship vs. all other specialties conditional on having completed rotation in that specialty as a medical student GEE *p* = 0.030

**Table 4:** Fellowship choice, mentorship and rotations

	Fellowship I intend to pursue *N* = 295	Specialty of Mentor in Residency^a^ *N* = 272	Rotations I rotated in during Medical School^b^ *N* = 415	Mandatory Rotations in Medical school^b^ *N* = 415
Cardiology	66 (22.4)	55 (20.2)	249 (60.0)	118 (28.4)
Gastroenterology	48 (16.3)	36 (13.2)	169 (40.7)	53 (12.8)
Hematology/Oncology	44 (14.9)	34 (12.5)	130 (31.3)	30 (7.2)
Pulmonary/Critical Care	43 (14.6)	40 (14.7)	249 (60.0)	145 (34.9)
Rheumatology	24 (8.1)	15 (5.5)	68 (16.4)	19 (4.6)
Nephrology	19 (6.4)	19 (7.0)	147 (35.4)	33 (8.0)
Endocrinology	11 (3.7)	6 (2.2)	86 (20.7)	28 (6.7)
General medicine	5 (1.7)	93 (34.2)	358 (86.3)	385 (92.8)
Infectious disease	13 (4.4)	21 (7.7)	165 (39.8)	33 (8.0)
Other	22 (7.5)	14 (5.1)		20 (4.8)

Statistics presented as N (column %)

^a^Could choose more than 1 mentor’s specialty

^b^Multiple rotations selected by each participant

Test of association between having a rotation in the specialty as a medical student and choosing the specialty in a fellowship among all 415 residents GEE *p* < 0.001

**Table 5:** Multivariable logistic regression of choosing Highly Sought After vs. Less Sought After Fellowships

*Effect*	OR (95% CI)	*P*-value
Age < 30 vs. > 30	0.94 (0.49, 1.77)	0.84
Female vs. Male	2.64 (1.47, 4.74)	0.001
White vs. non-White	0.69 (0.36, 1.30)	0.25
US vs. International Medical Graduates	0.88 (0.34, 2.25)	0.79
US Citizen vs. other	1.37 (0.50, 3.70)	0.54

### Resident perceptions of nephrology

**Fig. 1:**
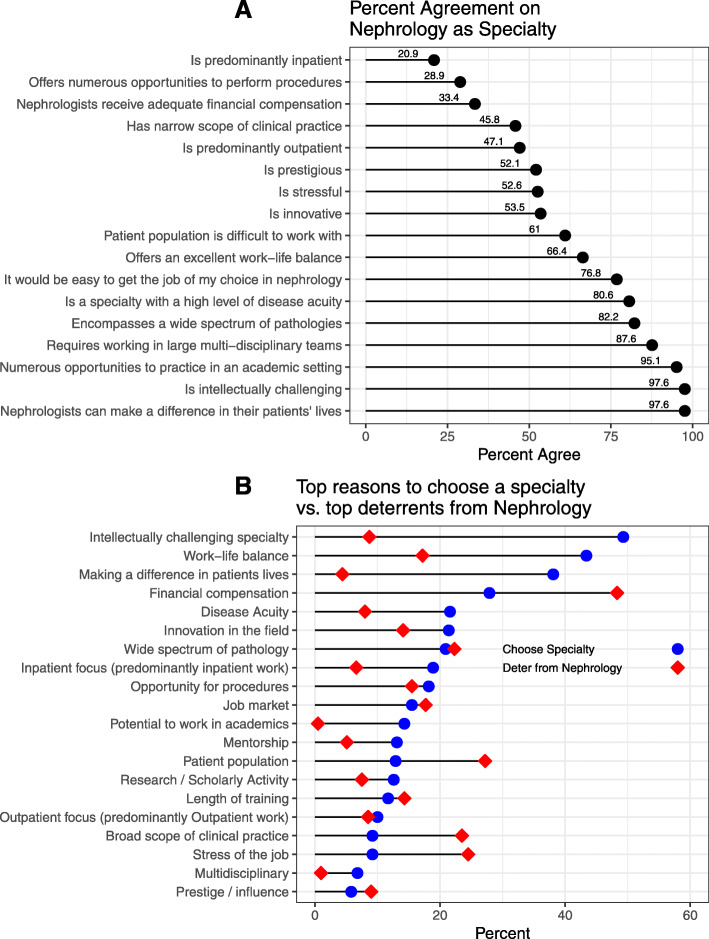
Residents’ perception of the nephrology specialty and factors influencing their career decisions. **a.** Displays residents’ perceptions of nephrology; residents were asked to relay their agreement to the corresponding statement relating to nephrology. **b.** Importance of factors promoting residents’ choice of a specialty generally (blue) and deterring them from pursuing nephrology specifically (red) displayed in percent

Respondents perceived nephrology positively in several areas: the specialty was perceived as intellectually challenging by 400 (97.5%) of respondents. The majority of residents 401 (97.6%) also believed that nephrologists had the ability to make a positive impact on their patients’ lives and 273 (66.4%) thought that nephrology offers a good work-life balance. On the other hand, several factors were perceived negatively in nephrology. For example, 273 (66.6%) of respondents indicated nephrologists did not receive adequate compensation while 293 (71.2%) noted that nephrology did not offer opportunities to perform procedures. The residents’ perceptions of nephrology are detailed in Fig. 1a.

### Factors influencing residents’ specialty choice

Residents were asked to identify the top factors that would influence their choice of a specialty (shown in Fig. 1b). The most frequently cited factors were intellectual challenge (49.3%), work-life balance (43.4%), ability to positively impact patients (38.1%), and financial compensation (27.9%). On the other hand, when asked to identify the top factors that would deter them from pursuing a fellowship in nephrology, they cited financial compensation (48%), patient population (27%), stress of the job (24.5%) and broad scope of clinical practice (23.4%).

**Fig. 2:**
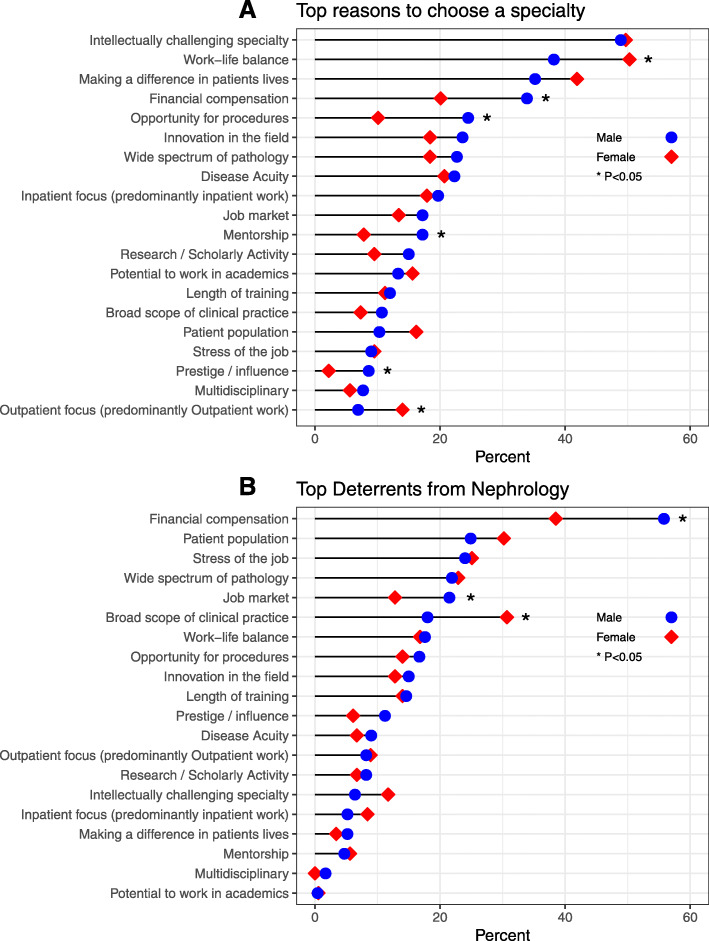
Factors influencing residents’ decision to pursue a specialty by gender. **a.** Shows the top factors that residents consider when pursuing a specialty. **b.** Shows the top factors that deter residents from pursuing nephrology. Male are represented in blue and females are represented in red

When comparing the top reasons to choose a specialty among genders, females had a significantly higher frequency of choosing work-life balance and significantly lower frequency of selecting financial compensation (shown in Fig. 2a). When comparing the top deterrents from nephrology among genders, females had a significantly lower frequency of choosing financial compensation as a deterrent (shown in Fig. 2b).

### Exposure to nephrology

During pre-clinical years of medical school, 340 (81.9%) respondents reported that nephrology was taught as an individual block. During clinical years of medical school, 33 (8%) of respondents reported that a nephrology rotation was mandatory. A total of 147 (35.4%) respondents rotated in nephrology. The relationship between medical school rotation and specialty choice is displayed in Table 3. Rotating in a specialty during clinical years of medical school was associated with choosing this specialty as a career in the future.

During residency, 141 (34.1%) of respondents reported that a nephrology rotation was mandatory but 317 (76.8%) rotated with a nephrologist. For 193 (46.5%) respondents, the nephrology rotation took place during PGY1 year vs. 177 (42.7%) in PGY2 and 74 (17.8%) in PGY3 training years. The majority of the nephrology interactions consisted of inpatient rotations: floor consults 239 (57.6%), 158 (38.1%) inpatient primary, 94 (22.7%) ICU nephrology, and 42 (10.1%) transplant service. 104 (25.1%) of responders rotated in ambulatory nephrology clinics.

### Quality of nephrology education

**Fig. 3:**
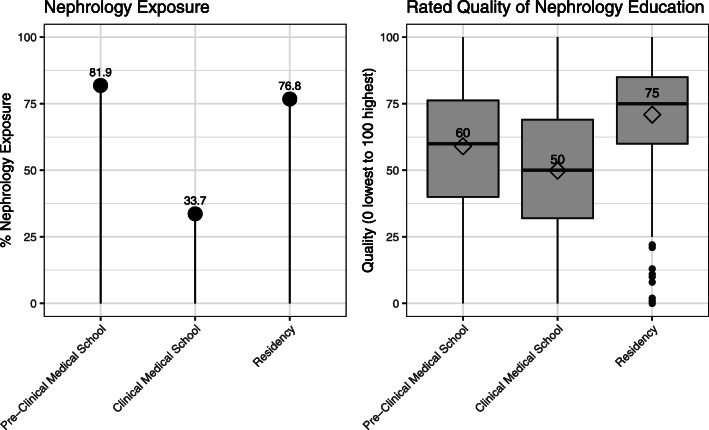
Exposure to Nephrology and quality of education during medical school and residency. Exposure to Nephrology during the pre-clinical years of medical school was defined as nephrology being taught as a separate individual block. Exposure to Nephrology during clinical years of medical school and residency was defined as a dedicated nephrology rotation ≥2 weeks long. Quality of nephrology education was rated on a scale of 1 (worst) to 100 (best)

The quality of nephrology education was rated on 0–100 scale by the respondents, with 100 being the best possible. It was rated positively during pre-clinical years of medical school (median 60) and during residency (median 75). It was rated less positively during clinical years of medical school (median 50) (shown in Fig. 3).

### Timing of specialty choice

Ten percent of respondents stated that they had decided on their choice of fellowship during the pre-clinical years of medical school versus 35% during the clinical years of medical school, 24.8% during PGY1, 21.4% during PGY2, 10% during PGY3 and 2.6% at another unspecified time.

### Mentorship

Sixty-six percent (*N* = 272) of responders had a mentor in the medical field. With the exception of general medicine, the mentor specialty appeared to mirror the fellowship of choice of the respondent (Table 4). Among residents that had a mentor, 19 (7%) had a mentor who was a nephrologist (Table 4).

## Discussion

The purpose of our study was to elucidate the factors that influence residents’ decision-making process in regard to their choice of specialty, and in particular, to understand the reasons driving them away from nephrology. There are three major findings in our study. 1) Female gender was the only factor associated with the choice of a less sought after fellowship. 2) Despite a positive perception of nephrology that aligns with reported resident priorities, three major deterrents appear to drive residents away from our specialty: perception of inadequate financial compensation, broad scope of the clinical practice, and a complex patient population. 3) There is a gap in both exposure to and quality of nephrology education during clinical years of medical school.

### Gender and choice of specialty

Gender was the only demographic factor that influenced the choice of specialty, as females chose less sought after fellowships more often than males (Table 5). Both males and females valued the intellectual challenge equally. However, females seemed to favor specialties that are predominantly ambulatory and offer a good work-life balance; while males favored procedural-based specialty with high prestige and high financial compensation (shown in Fig. 2a). It has long been postulated that specialties with a controllable lifestyle [13] are more appealing to females who may be trying to balance family and career responsibilities [14]. However, this approach is somewhat simplistic as gender inequity in medicine is a multifaceted issue that has been linked to gender bias and discrimination in the workplace [15]. Its complexity warrants a multilayered approach that our survey was not designed to evaluate.

None of the other demographic factors reported in Table 5 were associated with choosing a highly sought after specialty. Notably, and contrary to the general perception, the location of the medical school (United States vs. International) in our study was not associated with choosing a highly sought after fellowship. The number of USMGs interested in nephrology was noted to be low as early as 2009 [16] and has remained low since then. However, this seems to reflect a general decrease in nephrology interest rather than an issue related to the medical school per se.

### Why not nephrology? Analysis of resident perceptions

Overall, nephrology was perceived positively and aligned well with the top three factors influencing residents’ choice in a career specialty, namely: intellectual challenge, work-life balance and ability to make a difference in patients’ lives. However, in light of the profession’s struggle to fill its training positions, negative factors appear to outweigh the positives. Those negative factors consist in a perception of poor financial compensation, job stress, complex patient population, and broad scope of clinical practice (shown in Fig. 1a).

#### Not enough money or too much work?

Economic concerns are known to influence career choices of trainees [17] and low-income specialties tend to have lower fill rates than high-income specialties [18]. However, is this perception of inadequate nephrologist compensation true? According to the MedScape Physician Compensation report of 2020, the average annual compensation of nephrologists falls in the middle tier and in the same category as pulmonary-critical care and hematology-oncology [19]. Additionally, the ASN Adult Nephrology Workforce report shows that 72% of nephrologist aged over 55 reported their financial status to be “excellent” or “very good” as opposed to 5.5% considering their financial status as “fair” or “poor” [20]. This raises the question as to whether or not the problem is related to income per se rather than to Relative Value Unit (RVU), or in other words, adequate compensation for the amount of work done. The latter appears to tie into burnout. Indeed, burnout is becoming increasingly prevalent among nephrologists, with 49% of them reporting burnout symptoms and ranking third most severe among 24 other specialties [21]. The reasons for this burnout are numerous but have recently been linked to the restructuring of the health care system stripping away the three major pillars of intrinsic motivation: competence, relatedness and autonomy [22]. While all specialties face these issues, the profound lack of control over time and schedule seems to be particularly worse for nephrologists, whose heavy work load emphasizes frequent in-person visits to dialysis units [23, 24] thus requiring significant time and effort, notably driving from one dialysis unit to the other.

Given the known impact of burnout and the desire for a controllable lifestyle on the specialty choice of trainees [13, 25], we hypothesize that it is not the financial compensation per se that is driving residents away from nephrology, but rather the RVU of the work done. Indeed, it is widely believed that current RVUs are unfairly valued to favor procedure-based specialties over specialties requiring actual face time with complex patients, such as in nephrology [26]. This was clearly shown in a 2018 report for the Medicare payment advisory commission [27], which analyzed physician total cash compensation per work RVU, and found that nephrologists get less compensation per RVU than primary care physicians. Thus, despite a satisfying annual income, nephrologists need to work harder than their colleagues in other specialties.

#### Medical complexity or inadequate representation?

The majority of respondents reported finding the nephrology patient population challenging, and this is not surprising as nephrology patients are among the most medically complex in terms of number of co-morbidities, number of medications prescribed, and mortality risk [28]. In an Australian study, trainees even cited distress from negative patient interactions, particularly when caring for patients on dialysis [29]. While it is certainly true that nephrologists take care of dialysis patients, they also manage a multitude of intellectually challenging and stimulating pathologies in the ambulatory setting. However, the nephrology exposure of residents appeared to be largely limited to inpatient nephrology as only 32.8% of respondents reported an ambulatory nephrology experience. This could skew residents’ perception of the specialty, by exposing them to sick, potentially non-adherent dialysis patients. We hypothesize that this negative perception could be balanced by providing trainees with a more comprehensive nephrology exposure that includes more ambulatory interactions, including home dialysis and transplant patients [30].

#### Spectrum of clinical practice and the need to subspecialize

Another poorly recognized factor that could deter people from pursuing nephrology is the broad scope of the clinical practice. This has traditionally been perceived as a key attraction to nephrology with the underlying idea that nephrologists “never get bored” [31]. However, in our study, while the majority of our respondents recognized that nephrology has a broad scope of practice and offers a wide breadth of pathology (shown in Fig. 1a), they also cited those factors as deterring them from pursuing the profession (show in Fig. 1b). This appears to be in line with results reported in the rheumatology field, where a narrow practice scope was favored by the practicing physicians [32]. As the world of medicine continues to head into more advanced subspecialty, our results seem to suggest that nephrology may benefit from doing the same and developing more advanced subspecialty fields such as interventional nephrology, onco-nephrology, glomerular disease as examples.

### Exposure to nephrology and quality of education

Our findings unveil a possible gap in nephrology exposure and education (shown in Fig. 3). Indeed, both the quality and exposure appeared to be robust in pre-clinical years of medical school and in residency. However, during clinical years of medical school, we observed a decline in the number of students exposed to nephrology in general and in the perceived quality of that exposure. We suspect this decline to be relevant in steering careers away from nephrology. Indeed, clinical rotations have been shown to be among the most important factors determining career choices [33, 34]. This is consistent with our findings, which show that choosing a specialty is associated with rotating in this specialty, specifically during the clinical years of medical school (Table 3). One could argue that students are not choosing nephrology electives because they have already decided against it. However, the fact that only 10% of students in our sample decide on a specialty in their pre-clinical years argues against that hypothesis. Rather, a significant portion of respondents (35%, the largest) pursuing fellowship training reported deciding on their specialty choice during the clinical years of medical school. We believe this gap could represent an opportunity for improving the impact of nephrology on trainees and potentially reinvigorating the nephrology pipeline.

### Study strengths and limitations

The main strength of our study is its solid methodological design. First, this is one of the very few studies that is grounded in a well-established theoretical framework, which is crucial to substantiate the importance and significance of the work [35]. Second, to our knowledge, it is the only study that was preceded by a qualitative assessment, which allowed us to base our questions on the residents’ input rather than the investigators’ perceptions. Third, rather than focusing on nephrology physicians and fellows, our study focuses on internal medicine residents, and this population is of particular interest because these trainees constitute the main pipeline for the specialty.

Our study has several limitations. First, and though we reached out to all ACGME-accredited internal medicine programs in the US, only 26 agreed to participate in our survey. Thus, our sample was a sample of convenience. We also note that the majority of the respondents come from midwestern programs, and that could affect our representation of the national population and affect the external validity of our study. However, our respondents’ demographic characteristics appear to mirror those of internal medicine residents nationally, with 56.6% males in our dataset versus 57% males nationally, and 21.2% from international medical schools in our dataset vs. 23.1% nationally [36]. Our race and ethnicity distributions are also similar with a comparable representation of African Americans, Hispanics and Asians (respectively 5.1, 6.3 and 24.3% in our dataset versus 4.7, 6.7 and 24.1% nationally) [37]. Second, we recognize that our response rate of 21% is lower than the average 30% observed in studies involving medical clinicians [38] but it remains average for a web-based survey study. Indeed, web-based surveys usually yield a response rate that is on average 10% lower than mail surveys and ranges around 20–30% [39]. Furthermore, the number of nonrespondents does not correlate with the probability of nonresponse bias [40, 41]. Thus, our survey’s findings, while subject to the same limitations of survey designs, can be trusted to the extent of that design. Third, we recognize that we did not assess for the perceived quality of education of the other specialty and therefore cannot provided comparative data in that regard. Fourth, the RedCap survey platform displays clearly on mobile devices but the potential for incorrect responses still exists. Finally, despite a solid methodology along with numerous efforts to ensure the validity of the data collected, it is possible that some responses did not accurately measure the characteristics that we were seeking.

In summary, our study sheds light on factors that account for the declining interest in the nephrology specialty and identifies potential targets for improvement. Impacting some of those factors such as RVU-based compensation may require national policy changes; influencing other elements, such as moving the nephrology field towards sub-specialization, could be addressed at a systemic level with help from the different nephrology societies. Most importantly, as individual nephrologists, we have an opportunity to impact trainees’ perceptions of nephrology by improving their hands-on experience during the clinical years of medical school and broadening their exposure during residency to include more ambulatory settings. This requires our increased involvement in and commitment to the education of trainees. Future studies with larger and more representative sample size should be pursued to verify and improve our understanding of the current observations.

## Supplementary Information

**Additional file 1: Table S1.** Characteristics of Programs participating in the survey.

## Data Availability

The datasets generated and/or analyzed during the current study are available from the corresponding author on reasonable request.

## References

[1] National Resident Matching Program (2020). Results and data: specialties matching service. national resident matching program. published online.

[2] Pivert K (2019). Preliminary analysis—ASN data brief. ASN Data Analytics Published December 4.

[3] Saran R, Robinson B, Abbott KC (2017). US renal data system 2016 annual data Report: epidemiology of kidney disease in the United States. Am J Kidney Dis.

[4] Parker MG, Ibrahim T, Shaffer R, Rosner MH, Molitoris BA (2011). The future nephrology workforce: will there be one?. Clin J Am Soc Nephrol.

[5] Quigley L, Salsberg E, Colins A (2018). Report on the survey of 2018 nephrology fellow. American Society of Nephrology.

[6] Barat A, Goldacre MJ, Lambert TW (2018). Career choices for nephrology and factors influencing them: surveys of UK medical graduates. JRSM Open.

[7] Daniels MN, Maynard S, Porter I, Kincaid H, Jain D, Aslam N (2017). Career interest and perceptions of nephrology: a repeated cross-sectional survey of internal medicine residents. Barretti P, ed. PLoS One.

[8] Jhaveri KD, Sparks MA, Shah HH (2013). Why not nephrology? A survey of US internal medicine subspecialty fellows. Am J Kidney Dis.

[9] Nair D, Pivert KA, Baudy A, Thakar CV (2019). Perceptions of nephrology among medical students and internal medicine residents: a national survey among institutions with nephrology exposure. BMC Nephrol.

[10] Nakhoul GN, Mehdi A, Taliercio JJ (2020). Residents’ perception of the nephrology specialty. Kidney Int Rep.

[11] Cruess RL, Cruess SR, Boudreau JD, Snell L, Steinert Y (2015). A schematic representation of the professional identity formation and socialization of medical students and residents: a guide for medical educators. Acad Med.

[12] Hsieh H-F, Shannon SE (2005). Three approaches to qualitative content analysis. Qual Health Res.

[13] Schwartz RW, Haley JV, Williams C (1990). The controllable lifestyle factor and students’ attitude about specialty selection.16.Pdf. Acad Med.

[14] Lind DS (2003). Two decades of student career choice at the University of Florida: increasingly a lifestyle decision. Am Surg.

[15] Butkus R, Serchen J, Moyer DV, Bornstein SS, Hingle ST, for the Health and Public Policy Committee of the American College of Physicians (2018). Achieving gender equity in physician compensation and Career advancement: a position paper of the American College of Physicians. Ann Intern Med.

[16] Kohan DE, Rosenberg ME (2009). Nephrology training programs and applicants: a very good match. Clin J Am Soc Nephrol.

[17] Grayson MS, Newton DA, Thompson LF (2012). Payback time: the associations of debt and income with medical student career choice: student debt and career choice. Med Educ.

[18] Ebell MH (2008). Future salary and US residency fill rate revisited. JAMA..

[19] Kane L (2020). MedScape physician compensation Report.

[20] Salsberg E, Quigley L, Mehfoud N (2016). The US adult Nephrology Workforce: developments and trends.pdf. Published online.

[21] Martin KL (2020). MedScape physician lifestyle and happiness report.

[22] Hartzband P, Groopman J (2020). Physician burnout, Interrupted. N Engl J Med.

[23] Roberts JK (2018). Burnout in nephrology: implications on recruitment and the workforce. Clin J Am Soc Nephrol.

[24] Brady BM, Erickson KF (2019). Integrated care in ESKD: a perspective of nephrologists. Clin J Am Soc Nephrol.

[25] Dorsey ER, Jarjoura D, Rutecki GW (2003). Influence of controllable Lifestyle on recent trends in specialty choice by US medical students. JAMA..

[26] Rosner MH, Falk RJ (2020). Understanding work: moving beyond the RVU. Clin J Am Soc Nephrol.

[27] Zuckerman S, Shartzer A, Berenson R, Marks K, Das S, Brandt C. Analysis of Disparities in Physician Compensation. A report by the Urban Institute and SullivanCotter for the Medicare Payment Advisory Commission. December 2018. MedPac.gov. Published December 4, 2018. http://www.medpac.gov/docs/default-source/contractor-reports/jan19_medpac_disparities_physiciancompensationreport_cvr_contractor_sec.pdf?sfvrsn=0

[28] Tonelli M, Wiebe N, Manns BJ (2018). Comparison of the complexity of patients seen by different medical subspecialists in a universal health care system. JAMA Netw Open.

[29] Lane CA, Brown MA (2009). Nephrology: a specialty in need of resuscitation?. Kidney Int.

[30] Gomez AC, Warburton KM, Miller RK, Negoianu D, Cohen JB (2017). An interactive ambulatory nephrology curriculum for internal medicine interns: design, implementation, and participant feedback. Am J Kidney Dis.

[31] Beckwith H, Kingsbury M, Horsburgh J (2018). Why do people choose nephrology? Identifying positive motivators to aid recruitment and retention. Clin Kidney J.

[32] Zborovski S, Rohekar G, Rohekar S (2010). Strategies to improve recruitment into rheumatology: results of the workforce in rheumatology issues study (WRIST). J Rheumatol.

[33] Kolasinski SL, Bass AR, Kane-Wanger GF, Libman BS, Sandorfi N, Utset T (2007). Subspecialty choice: why did you become a rheumatologist?. Arthritis Rheum.

[34] Blachman NL, Blaum CS, Zabar S (2019). Reasons geriatrics fellows choose geriatrics as a career, and implications for workforce recruitment. Gerontol Geriatr Educ.

[35] Lederman NG, Lederman JS (2015). What is a theoretical framework? A practical answer. J Sci Teach Educ.

[36] AAMC resident report 2020 – number of active residents by type of medical school, GME specialty and sex. AAMC Data Reports. Accessed January 15, 2021. https://www.aamc.org/data-reports/students-residents/interactive-data/report-residents/2020/table-b3-number-active-residents-type-medical-school-gme-specialty-and-sex

[37] AAMC report 2020 - number of active MD residents by race / ethnicity and GME specialty. AAMC Data Reports. Accessed January 15, 2021. https://www.aamc.org/data-reports/students-residents/interactive-data/report-residents/2020/table-b5-md-residents-race-ethnicity-and-specialty

[38] Ma IG-G (2008). Effects of various methodologic strategies. Can Fam Physician.

[39] Shih T-H, Fan X (2008). Comparing response rates from web and mail surveys: a meta-analysis. Field Methods.

[40] Groves RM (2006). Nonresponse rates and nonresponse Bias in household surveys. Public Opin Q.

[41] Halbesleben JRB, Whitman MV (2013). Evaluating Survey Quality in Health Services Research: A Decision Framework for Assessing Nonresponse Bias. Health Serv Res.

