# Supplementary material for: “What do you think about nephrology?” A national survey of internal medicine residents
Source: BMC Nephrol. 2021 May 21;22:190. doi: 10.1186/s12882-021-02397-9 (PMC8140430; doi:10.1186/s12882-021-02397-9)
Supplement: Supplementary file 1 — Additional file 1: Table S1. Characteristics of Programs participating in the survey. [file 12882_2021_2397_MOESM1_ESM.doc]

**Appendix Table 1. Characteristics of Programs participating in the survey**

|  | **Total (N=26)** |
| --- | --- |
| N residents in program, Median [P25, P75] | 67.5[36.0,101.0] |
| Program size |  |
| < 46 residents | 10(38.5) |
| 46-75 residents | 6(23.1) |
| > 75 residents | 10(38.5) |
| Program type |  |
| Community based | 11(42.3) |
| University based | 15(57.7) |
| Tertiary referral center |  |
| Yes | 24(92.3) |
| No | 1(3.8) |
| Unsure | 1(3.8) |

Statistics shown as N (column %) unless otherwise noted
